# Ketamine’s Therapeutic Role in Substance Use Disorders: A Narrative Review

**DOI:** 10.3390/neurosci6030083

**Published:** 2025-08-27

**Authors:** Alexander Thomas, R. Andrew Chambers

**Affiliations:** Indiana University School of Medicine Addiction Psychiatry Fellowship Program, Goodman Hall, Department of Psychiatry, Indianapolis, IN 46202, USA; thomaals@iu.edu

**Keywords:** ketamine, SUDs (substance use disorders), review, NMDA (N-methyl-D-aspartate), controlled trial, cohort studies, case reports, retrospective studies, neuroscience, treatment

## Abstract

Interest in ketamine as a novel treatment for substance use disorders (SUDs) has been increasing due to its N-methyl-D-aspartate (NMDA) glutamate receptor antagonism and mounting evidence that glutamate neurotransmission is involved in the pathogenesis of both depression and addictions. This narrative review provides an outline of clinical evidence reported in the literature from the 1970s to 2025 that examines the efficacy of ketamine for the treatment of SUDs, focusing primarily on randomized blinded controlled trials (RBCTs). Key cohort studies, retrospective studies, secondary analyses, case reports, and relevant basic neuroscience studies are reviewed to complement the more rigorous human controlled trial data. Thus far, ketamine has been tested in nine RBCTs targeting cocaine (three studies), alcohol (three studies), opioid use disorder (two studies), and nicotine (one study), suggesting efficacy for addiction in combination with psychotherapies, and often when doses produce subjectively reported mystical or psychedelic experiences. This review highlights promising preliminary evidence, and the need for more rigorous studies to elucidate the scope of drug addictions ketamine may target, its optimal dosing or route of administration, the importance of concurrent psychotherapies, professional supervision and safety monitoring, and which psychiatric comorbidities or contexts may contraindicate its use for SUDs.

## 1. Introduction

Ketamine, first called CI-581, was originally synthesized in 1962 by Calvin L. Stevens as a replacement for PCP as a general anesthetic [1]. Ketamine has a good safety profile notably with a lack of respiratory suppression. Thus, it can be used without electricity or supplemental oxygen and has been deemed an essential medicine by the World Health Organization [2]. Currently, it is used for recreational or medical purposes across four general dose ranges: sub-psychedelic (“K-land”), medium or high psychedelic (“K-hole”), and as a general anesthetic [3]. Due to its acute psychotogenic effects, ketamine has also been used as a pharmacologic model of schizophrenia in both animal models and humans [4,5,6,7]. Within this context, it was serendipitously discovered that ketamine has efficacy for treatment-resistant major depression [8], leading to the 2019 FDA approval of the intranasal formulation of ketamine’s s-enantiomer, esketamine, to be delivered under medical supervision.

Ketamine’s history as a candidate for the treatment of substance use disorders (SUDs) actually predates its characterization as a treatment for depression in the U.S. In Mexico, Dr. Salvador Roquet used ketamine and other psychedelics to augment psychotherapy for the treatment of SUDs and other psychiatric illnesses from 1964 to 1974, which ultimately led to his incarceration [9]. Subsequently, in Russia, Dr. Evgeny Krupitsky published his review of ketamine psychedelic therapy in 1997 reporting on his open label treatment of over one thousand patients with alcohol use disorder [10].

More recently, a handful of controlled trails testing ketamine for the treatment of SUDs have been conducted [11]. Concurrently with this growing clinical literature, and as inspired by ketamine’s success as a treatment for depression, a growing body of basic science has begun to explore the potential mechanisms by which ketamine may produce therapeutic change in depression and SUDs (see Ezquerra-Romano et al. from 2018) [12]. Ketamine has complex and wide-ranging effects on receptors and cellular processes. In addition to being a non-competitive NMDA receptor antagonist, it blocks nicotinic acetyl-choline ion channels, has delta and mu-opioid agonism and potentiation, interacts with the nitric-oxide and cyclic guanosine-mono-phosphate system, causes AMPA upregulation, and increases release of dopamine and noradrenaline [13].

Since the initial 2000 study by Berman et al. [8] demonstrated ketamine’s antidepressant properties, a plethora of rigorously designed studies have been conducted demonstrating its efficacy for treating major depressive disorder and suicidal ideation [14]. As evidenced by the capacity of ketamine to decrease functional connectivity between the Default Mode Network and the medical prefrontal cortex, which may be increased in the context of major depression, ketamine may produce beneficial effects on the scale of neural networks that are “stuck” in psychiatric illness [15]. Thus, ketamine may be effective for a wide range of mental illnesses that are commonly comorbid with depression, including social anxiety disorder, generalized anxiety disorder, obsessive–compulsive disorder, posttraumatic stress disorder, and eating disorders [14].

Since the neurocircuitry underpinning major depression and many of these other mental illnesses are inter-connected and casually inter-related with SUDs [16,17], it stands to reason that circuits and neurotransmitter systems targeted by ketamine for mental illness may also address SUDs. Alterations in glutamatergic signaling corresponding to impaired neuroplasticity and neurogenesis are implicated in both depression and SUDs [18,19,20]. Ketamine administration may reverse these dynamics by increasing synaptogenesis through activation of the mammalian target of rapamycin (mTOR) pathway [21]. Ketamine administration also increases Brain-derived neurotrophic factor (BDNF) [22], which may be decreased in the brains of patients with SUDs [23].

Ketamine’s effects on altering or increasing extracellular glutamate levels may also therapeutically benefit corticolimbic system connectivity disruptions that keep patients stuck in SUDs [24,25]. These dynamics could reflect neuroinformatic (i.e., how neural networks store and process information representations) impacts of ketamine. Memories stored in the brain, including those that govern motivational control, are not fixed and permanently hardwired but instead are transiently modified and re-encoded over many episodes of retrieval and reconsolidation [26,27]. Disruption of reconsolidation processes via ketamine administration is thus a plausible mechanism by which it may weaken maladaptive drug-reward memories and drug reinforcement [28].

The psychedelic effects of ketamine including hallucinations, mystical experiences, and ‘near-death’ or ‘out-of-body’ experiences that people may experience during acute intoxication may represent subjective correlates of the acute neuroinformatic effects of the drug that are proposed to have therapeutic potential [29]. To the extent that mystical like experience with ketamine may also reflect a neuroplastic impact of the drug, ketamine has been posited to enhance the efficacy of psychotherapy and promotion of learning new behaviors (e.g., as in recovery from SUDS) [3].

Despite the significant societal costs associated with SUDs and urgency associated with addressing the addiction epidemic, NMDA receptor antagonists such as ketamine remain relatively unexplored for SUD indications. Here, we provide an updated narrative review of current evidence in support of this treatment possibility that builds on other recent prior reviews [30,31], while highlighting the strongest clinical evidence (e.g., randomized controlled trials) relevant to different forms of addictive drugs. This review also highlights key emerging themes of translational neuroscientific importance to this rapidly growing area of investigation, such as whether psychotherapeutic treatments and/or psychedelic experiences occurring concurrent with ketamine treatment may be key for its therapeutic potential in SUDS. In constructing this narrative review, PubMed and Google Scholar searches were conducted up to 28 June 2025 for English language, peer-reviewed manuscripts containing the terms ketamine, alcohol, opioid, opiate, cocaine, methamphetamine, nicotine, tobacco, substance use disorder, and/or addiction. Top priority was given to incorporating all existing randomized blinded control trials (RBCTs), including studies that tested ketamine as a primary intervention for the treatment of a given type of SUD. Primary controlled trials, exploratory clinical studies and existing review articles found in the initial search underwent secondary bibliography searches to capture informative cohort studies, case reports, retrospective studies, and preclinical investigations (particularly when in the absence of human evidence for a given addictive drug type). No integrative statistical assessment of the clinical evidence or rigorous assessment of bias was carried out for the purpose of this review. The characteristics of seven double-blind (RBCTs) examining ketamine for SUDs published by June 2025 are summarized in Table 1, with other key studies including two single-blind RBCTs summarized in Table 2.

### 1.1. Alcohol Use Disorder

The first published study using ketamine to treat SUDs is from Russia in 1992. This randomized trial involved 186 men with alcoholism who failed aversive emetic therapy (administering nausea-inducing drugs during alcohol consumption), pharmacological treatment, and individual and group therapy for three months. They either continued treatment as usual or underwent treatment with ketamine. Eighty-six men were randomized to receive ketamine treatment in three stages: an explanation of the procedure, the ketamine administration itself, and group psychotherapy. Notably, during ketamine (3 mg/kg IM) administration, patients smelled and tasted alcohol during the curated, aversive psychedelic experience. At the 1-year follow-up, 60/86 (69.8%) of patients assigned to the ketamine treatment group reported abstinence from alcohol as compared to 24/100 (24%) of the control group. Ketamine treatment was more effective than all other forms of treatment at that time and there were no stated adverse reactions during ketamine administration [43].

A 1997 prospective cohort study and review of underlying mechanisms of ketamine administration for the treatment of alcoholism expanded on these findings. Two hundred and eleven men with alcoholism were able to choose between ketamine treatment or conventional psychotherapy after 3 months of individual and group psychotherapy. In total, 111 chose ketamine treatment carried out in three stages: an explanation alongside treatment of psychiatric comorbidities, ketamine administration, and group psychotherapy. Ketamine (2.5 mg/kg IM) was administered alongside a curated, aversive experience. Measured by self-report, 73/111 (65.8%) of the ketamine treatment group attained one or more years of sobriety as compared to 24/100 (24%) of the control group. At the 2-year follow-up and 3-year follow-up for the ketamine group, 33/81 (40.7%) and 14/42 (33.3%), respectively, maintained sobriety [10]. The authors conclude by stating that they successfully carried out ketamine treatment on over 1000 patients without any complications including ketamine use disorder [10].

Inspired by Krupitsky, Kolp et al. conducted a retrospectively reported case series in the U.S. of 70 adults with alcohol dependence treated with ketamine-enhanced psychotherapy, which is described as psychoanalytic therapy focusing on ketamine’s ability to replicate near-death experiences. Based on their best recollection, they estimate up to 90% of patients had a comorbid SUD and 50% with a psychiatric comorbidity. Initially, ketamine-enhanced psychotherapy was offered as part of an outpatient weekly ten-session treatment program where ketamine (unknown dose) was administered on the seventh session and the transpersonal experience was subsequently integrated. This approach led to an approximately 25% abstinence rate at 1 year. In an attempt to replicate Krupitsky’s 70% abstinence rate at one year, ketamine-enhanced psychotherapy went through four iterations and was ultimately a 3-week-long program with 90 h of psychotherapy weekly and two ketamine sessions. Within this context, patients achieved the 70% abstinence rate at 1 year [44].

Das et al. conducted a single-blind randomized controlled trial of 90 non-treatment-seeking adults with hazardous/harmful drinking patterns without alcohol use disorder (AUD) to either receive (1) retrieval/destabilization of maladaptive alcohol memories (RET) and ketamine, (2) no RET (RET procedure for non-drinking memories) and ketamine, or (3) RET and placebo to test if ketamine can disrupt the reconsolidation of maladaptive reward memories (MRMs). RET or no RET was performed and 5 min later, an IV ketamine infusion (serum concentration 350 ng/mL for 30 min) or IV saline placebo infusion began. One week later, participants that received RET and ketamine had significantly decreased urges to drink, decreased enjoyment of alcohol, and decreased number of binges. None of these differences were observed in the control groups. Nine months later, RET and ketamine showed the greatest reduction in total alcohol consumption. There was a significant correlation between ketamine and metabolite levels during the period of memory instability after RET but not for no RET, suggesting that ketamine had a dose-dependent relationship to reconsolidation blockade. The authors conclude that ketamine can interfere with the reconsolidation of maladaptive alcohol memories and thus reduce drinking in this population [28].

An open-label pilot study recruited five patients with major depressive disorder (MDD) and comorbid AUD to receive injectable naltrexone 2–6 days before the initiation of weekly ketamine infusions (0.5 mg/kg IV) for four weeks. All showed a 50% or higher improvement from baseline in the Montgomery–Asberg Depression Rating Scale by 4 weeks and 4/5 (80%) reported improvement in alcohol craving as measured by the Obsessive Compulsive Drinking Scale. The authors conclude that naltrexone did not interfere with the clinical response to ketamine and that ketamine treatment may enhance the treatment of comorbid AUD by decreasing cravings [45].

A randomized, double-blind, active control, pilot trial explored the effects of a single ketamine infusion (0.71 mg/kg IV over 52 min) vs midazolam (0.025 mg/kg IV over 52 min) and motivational enhancement therapy (MET) for the treatment of AUD. Forty treatment-seeking adults with alcohol dependence either received ketamine (17/40) or midazolam (23/40) during the second week of a five-week outpatient trial. Both groups received a total of six MET sessions. Measured by self-report and urine toxicology, patients who received ketamine had a significantly higher proportion of alcohol-abstinent days during the last 3 weeks of the study. During this time, the proportion of heavy drinking days increased in the midazolam group but remained stable in the ketamine group and time to relapse was significantly higher in the ketamine group. At the 6-month follow-up, via telephone interviews, 6/8 (75%) of the ketamine group reported abstinence compared to 3/11 (27%) in the midazolam group. There were no persistent psychoactive effects or initiation of drug misuse in either study group [32].

Secondary analysis of this study examined the mystical experience via the Hood Mysticism Scale (HMS) and the dissociative effects as measured by the Clinician Administered Dissociative States Scale (CADSS). Ketamine produced significantly higher scores on both scales as compared to midazolam. The HMS had a significant correlation with decreased heavy drinking days, but the CADSS did not. Similarly, increased HMS score was significantly correlated with longer time to relapse, but CADSS was not [46].

Grabski et al. conducted a randomized, double-blind, placebo-controlled phase 2 clinical trial with 96 adults with moderate to severe AUD. These adults were randomized to one of four treatments: (1) ketamine and mindfulness-based relapse prevention therapy, (2) ketamine and alcohol education, (3) saline and therapy, or (4) saline and education in a 1:1:1:1 ratio. Ketamine (0.8 mg/kg IV over 40 min) or placebo (0.9% IV saline of equal volume) were administered at visits 2, 4, and 6. Therapy or education was given on visits 2–8. Using the Alcohol Timeline Follow Back self-report questionnaire at the 3- and 6-month follow-ups, there was a significantly greater percentage of days of abstinence in the ketamine and therapy group as compared to saline and education. However, there was no significant difference for odds of relapse within 6 months. Six participants reported using ketamine illicitly on a single occasion after receiving the infusions but all of them had previously used ketamine recreationally [33].

Terasaki et al. conducted a three-arm, open-label randomized trial comparing the effect of (1) a single infusion of ketamine (0.5 mg/kg IV over 40 min) and linkage to an outpatient addiction clinic, (2) naltrexone (380 mg IM) and linkage, or (3) linkage alone on 30-day all-cause hospital readmission rates. Forty-four hospitalized adults with severe AUD and at least one alcohol-related hospitalization or emergency department visit within the past year were randomized. On the anticipated day of discharge, the intervention occurred without psychotherapy. The 30-day readmission rates were 2/13 (15.4%) for ketamine, 3/14 (21.4%) for naltrexone, and 7/17 (41.2%) for linkage alone. Clinic attendance was 8/13 (61.5%) for ketamine, 7/14 (50%) for naltrexone, and 7/17 (41.2%) for linkage alone. However, these outcomes were not statistically significant, which the authors assert is due to their small sample size. There were no serious adverse events or reported illicit ketamine use [39].

### 1.2. Alcohol Withdrawal

Wong et al. looked at a retrospective cohort of 23 adults admitted to the ICU that received an adjunctive ketamine infusion (mean total infusion rate 0.20 mg/kg/h IV for approximately 56 h) for the management of alcohol withdrawal syndrome; usually delirium tremens. Comparing pre- and post-administration, they found ketamine may reduce benzodiazepines requirements without affecting sedation scores [47]. Pizon et al. conducted a retrospective cohort study of 63 adult patients admitted to ICU diagnosed with delirium tremens. As compared to those treated with solely symptom-triggered benzodiazepines and phenobarbital, ketamine infusion (mean 0.19 mg/kg/h for approximately 47 h) was associated with a significant decrease in the length of ICU stay, likelihood of intubation, and mean benzodiazepine dose received [48]. Shah et al. conducted a retrospective cohort study of 30 adults admitted to the ICU that received adjunctive ketamine (median 0.75 mg/kg/h for approximately 54 h) for lorazepam infusion-resistant alcohol withdrawal syndrome. Initial symptom control was achieved within 1 h for all patients alongside a significant reduction in benzodiazepine requirements [49]. Combining the above studies, the only adverse effect reported was oversedation in two patients.

### 1.3. Opioid Use Disorder

Ketamine’s treatment of opioid use disorder (OUD) is multifaceted. There are numerous studies demonstrating that ketamine can reduce both acute and chronic pain [50,51,52] and reduce the respiratory suppression and hyperalgesia associated with opioid use [51]. Taken together, this evidence suggests ketamine may reduce the total amount of opioids required for adequate pain control, potentially decreasing the incidence of OUD from opioid exposure.

Ketamine can be used to treat OUD directly. A 2002 double-blind, randomized controlled trial in Russia compared the effects of a psychedelic dose of ketamine (2.0 mg/kg IM) with a non-psychedelic dose (0.2 mg/kg IM) in an inpatient setting. This included psychotherapy in preparation, during ketamine administration, and afterwards. Seventy detoxified heroin-addicted patients were randomly assigned in a 1:1 ratio. Measured by self-report, collateral information, and urine toxicology, the psychedelic dosing produced a significant increase in abstinence and lower cravings at 24 months. Both groups had decreased anhedonia, anxiety, and depression alongside improvements in internal locus of control, understanding meaning and purpose of one’s life, spirituality, and attitude towards abstinence. There were no psychiatric complications including the development of ketamine use disorder and this intervention proved more effective than conventional treatments at the time [34].

A follow-up randomized clinical trial by the same group in Russia compared three ketamine sessions with a single session. Each group received the same psychedelic dose of ketamine (2.0 mg/kg IM). Fifty-nine detoxified patients with heroin dependence were randomly assigned to each group. Like the prior study, patients received psychotherapy before, during, and after their first ketamine session. Both groups received monthly 1-h addiction counseling sessions with the three-session group having two additional ketamine sessions following counseling. At 1 year follow-up, the rate of abstinence in the three-session group was significantly higher, 13/26 (50%), as compared to 6/27 (22.2%). The additional improvements noted in their 2002 study were seen alongside no differences in cravings, anxiety, or depression [40].

An open-label proof of concept study examined the combination of ketamine, rTMS, and trauma interventions using mindfulness-based extinction and reconsolidation of memories (TIMBER). This study included three patients with a diagnosis of OUD that underwent a single infusion of ketamine (0.75 mg/kg IV over 45 min) and, 1 week later, five sessions of rTMS to the right dorsolateral prefrontal cortex alongside five sessions of TIMBER over 1 to 2 weeks. There was a significant decrease in every patient’s Opiate Craving Scale score [53].

A randomized, double-blind, double-arm, active control study in Iran compared one treatment with ketamine (0.5 mg/kg over 40 min) or buprenorphine (16 mg sublingual) for anxiety and opioid cravings measured by the HAM-A and Opioid Cravings Scale for 64 inpatient adult participants with comorbid MDD and OUD. Both interventions led to significant decreases in anxiety and opioid craving at 2 h, 24 h, and 1 week post-intervention without differences between the two groups [35].

### 1.4. Opioid Withdrawal

A randomized, placebo-controlled, double-blind trial in Lithuania compared normal saline or a subanesthetic ketamine infusion (0.5 mg/kg/h IV) for management of opioid withdrawal during rapid opioid antagonist induction under general anesthesia. Fifty-eight patients with opiate dependence were admitted and stabilized on intramuscular morphine. On day 3, patients were put under general anesthesia and received naloxone and naltrexone. Of the 50 patients included in the final analysis, those that received the ketamine infusion had significant suppression of precipitated withdrawal as evidenced by an attenuated rise in cardiovascular, respiratory, and neuroendocrine response [54].

Heeny et al. conducted a retrospective case series involving 10 patients with buprenorphine-precipitated withdrawal treated with ketamine. They were generally given a ketamine infusion (0.3 mg/kg IV over 15 min) followed by an addition infusion (0.3–1 mg/kg over 1 h). All of these patients had reported improvement in their clinical opioid withdrawal scale (COWS) [55]. Some other examples include Hailozian et al. who presents a case of a fentanyl-using patient with OUD who developed severe buprenorphine-precipitated withdrawal during an outpatient buprenorphine micro-induction that was successfully treated by a ketamine infusion (0.6 mg/kg IV over 1 h) [56]. Christian et al. present a case of a patient with OUD who developed severe buprenorphine precipitated withdrawal with a COWS > 36 after presenting to the emergency department and receiving buprenorphine. He was treated with ketamine (0.6 mg/kg IV over 75 min on two separate occasions) and withdrawal resolved [57]. Our own addiction psychiatry clinic has reported a case of a patient with OUD, MDD, OCD, and chronic pain who spontaneously (without medical recommendations) tapered down their buprenorphine/naloxone dosing from 16 mg to 8 mg a day without experiencing withdrawal or rebound craving during the early stages of esketamine treatment [58]. Within this context, sublingual, sub-dissociative dosed ketamine has been studied in a polit case series to transition patients from fentanyl and methadone to buprenorphine, with the majority of 37 patients reporting either significant improvement or resolution of precipitated withdrawal [59].

### 1.5. Cocaine Use Disorder

A 2014 randomized, double-blind, lorazepam-controlled, crossover trial by Dakwar et al. examined the effects of three infusions, two different doses of ketamine (0.41 mg/kg IV and 0.71 mg/kg IV over 52 min) vs. lorazepam (2 mg IV over 52 min), on cue-induced cravings measured by the visual analog scale for cocaine craving and motivation to quit cocaine measured by the University of Rhode Island Change Assessment. Eight non-treatment-seeking patients with cocaine dependence received each infusion separated by 48 h and outcomes were assessed 24 h post-infusion while admitted to a research unit. Both doses increased motivation to quit and decreased cue-induced craving but the higher dose further reduced cue-induced craving. At 4 weeks, measured by Timeline Follow Back and urine toxicology, there was a significant decrease in the amount and frequency of cocaine use in the ketamine group. There were no adverse psychiatric effects and no initiation of ketamine or benzodiazepine misuse [36].

Secondary analysis of this trial explored if the mystical experiences from ketamine infusions influenced the observed effects. Using a modified version of the HMS, ketamine led to significantly higher scores than the lorazepam infusion, with the higher dose of ketamine being the highest. They found that the intensity of the mystical experience mediated the motivation to quit cocaine at 24 h while CADSS did not [60].

A follow-up randomized, double-blind, midazolam-controlled, crossover trial by the same group looked at the effect of a single ketamine infusion (0.71 mg/kg IV over 52 min) vs. midazolam (0.025 mg/kg IV over 52 min) on cocaine self-administration. Twenty non-depressed, non-treatment-seeking patients with cocaine dependence were admitted to a research unit for 6 days three times to analyze the effects of (1) normal saline, (2) ketamine, and (3) midazolam with normal saline first and randomly ketamine or midazolam second and third. A 70-min choice session consisting of five choices between cocaine and money was conducted 28 h post-infusion. Ketamine led to a significant reduction in cocaine choices (1.61/5) as compared to both saline baseline (almost 5/5) and midazolam (4.33/5). Notably, two patients entered remission from cocaine use after ketamine administration and were thus ineligible for the third hospitalization. There were no notable adverse effects [37].

Secondary analysis of the above study explored the mystical experiences and if this mediated ketamine’s observed effects. The CADSS, the HMS, and the Near-Death Experiences Scale (NDES) were assessed. Ketamine led to significantly higher psychoactive effects on all measures compared to midazolam. The decreases in cocaine self-administration and cravings were mediated by the HMS but not the CADSS or NDES. The authors noted the psychoactive effects were transient and well tolerated [61].

These studies culminated in a randomized, double-blind, midazolam-controlled, clinical trial that compared one ketamine infusion (0.5 mg/kg IV over 40 min) to an infusion of midazolam (0.025 mg/kg IV over 40 min) with 5 days of mindfulness-based relapse prevention (MBRP) in the inpatient setting. Fifty-five treatment-seeking patients with cocaine dependence were randomized. All received MBRP daily on days 2–5 and their infusion occurred earlier on day 2. They then returned twice weekly for 4 additional weeks of MBRP. During the last 2 weeks of the trial, 13/27 (48.2%) of the ketamine group and 3/28 (10.7%) of the midazolam group had urine-test-confirmed abstinence from cocaine. At the 6-month follow-up, 12/27 (44%) of the ketamine group self-reported abstinence from cocaine compared to 0/28 (0%) of the midazolam group. Additionally, the ketamine group had lower levels of cocaine craving scores and a longer time to relapse. No adverse psychiatric effects or development of substance misuse were noted [38].

A retrospective cohort study used artificial intelligence (AI) prediction and an expert panel to determine candidates for the treatment of cocaine use disorder and their potential mechanisms of action. The AI identified 35 drug candidates and ketamine was selected by the expert panel for further analysis. The AI compared patients with cocaine use disorder that were either exposed to ketamine as a general anesthetic or for the treatment of depression to controls. After analysis of over 90 million patients from the U.S., patients with ketamine exposure were more likely to achieve remission than controls (HR 1.98 for ketamine as a general anesthetic and 4.39 for ketamine as an antidepressant). It also identified that ketamine targets multiple genes of interest that are also implicated in AUD, stimulant use disorder, OUD, and nicotine use disorder [62].

### 1.6. Cannabis Use Disorder

A single-medication-blind proof of concept study by Azhari et al. investigated subanesthetic ketamine combined with motivation enhancement therapy (MET) and MBRP for the treatment of cannabis use disorder over 6 weeks. Eight treatment-seeking patients with cannabis dependence received one ketamine infusion (0.71 mg/kg IV over 52 min) during week 2 after 24 h of abstinence and non-responders received a second infusion (1.41 mg/kg over 92 min) during week 4. Mindfulness-based exercises were used during each infusion. They also received MET on week 1, day 1, MET on the day before their first ketamine infusion, MET the afternoon after the infusion, and MBRP twice weekly on weeks 3 to 6. The non-responders, 3/8, as defined as continued use or intolerable cravings, received MET the day before the second infusion and the afternoon after. There was a significant decrease in the number of cannabis-using days measured by Timeline Follow Back starting 1 week after the first infusion and lasting through the end of the study. There was an increase in patients’ confidence to abstain from using cannabis but no differences were found for craving. Overall, 6/8 participants achieved 3 or more weeks of abstinence confirmed by urine toxicology by the end of the study. No participants developed misuse of any other substance and no adverse psychiatric effects were noted [41].

### 1.7. Tobacco Use Disorder

A small, randomized, single-blind, placebo-controlled pilot study by Chuang et al. investigated a single infusion of subanesthetic ketamine without psychotherapy for the treatment of tobacco use disorder over 8 days in a non-treatment-seeking sample. Ten participants were randomized and six received a single infusion of ketamine (0.5 mg/kg over 20 min) on day 1 in addition to completing a 7-day smoking diary pre- and post-infusion. The ketamine infusion was safe and well tolerated. There was a nonsignificant decrease in the Questionnaire of Smoking Urges, potentially indicating lower cravings, and there was a nonsignificant trend towards a decrease in the Minnesota Withdrawal Scale. There was no significant effect on self-reported cigarette use [42].

### 1.8. Methamphetamine Use Disorder

We know of no published studies that examine ketamine for the treatment of methamphetamine use disorder, but this is an area of ongoing research (CTN-0132). However, a preclinical study demonstrated potential benefit. Sprague-Dawley rats were given methylazoxymethanol (MAM) acetate in accordance with the MAM rat model of schizophrenia or saline. Seven control rats and five MAM rats were allowed to self-administer methamphetamine (0.08 mg/kg/infusion IV) for a 14-day maintenance period followed by a 14-day abstinence period. Then, a 5-day relapse period to re-establish drug taking behavior with a saline intraperitoneal injection to assess the effect on methamphetamine self-administration. At least one day after this, ketamine (5 mg/kg IP) was administered. There was no significant change in self-administration of methamphetamine in MAM rats after ketamine administration. However, there was a significant decrease in the amount of methamphetamine self-administered in control rats [63].

## 2. Discussion

The evidence reviewed here, anchored on seven double-blind RBCTs, has characterized ketamine’s use for cocaine (three studies), alcohol (two studies), and opioid use disorder (two studies). Two additional single-blind randomized controlled trials have examined ketamine treatment in alcohol and nicotine use disorder, while there is a lack or rigorous controlled studies for stimulant or cannabis use disorders. Evidence from the AUD literature points to ketamine improving SUD outcomes while treating psychiatric comorbidities [45], disrupting maladaptive reward memories [28], and providing beneficial mystical experiences [10,43,46]. Similarly, evidence from the OUD literature supports improvement through treating psychiatric comorbidities [35] and providing beneficial mystical experiences [34,40]. The cocaine use disorder (CUD) literature further supports the importance of mystical experiences [60,61]. Taken together, these all represent clues about ketamine’s possible mechanisms of action and its utility in the treatment of SUDs [10,28,34,35,40,43,45,46,60,61]. Based on the AUD, OUD, and CUD literature, subanesthetic ketamine administration may reduce key symptoms of SUDs, manifesting subjectively as decreased cravings [28,34,36,38,40,45,53] or, also including the tobacco use disorder literature, more objectively as drug abstinence [10,32,33,37,42,43,44]. Moreover, the AUD, OUD, and CUD treatment evidence suggests ketamine may produce long-lasting effects for SUDs that far surpass the duration of drug delivery (on the order or weeks or months) [10,32,33,34,38,40,43,44].

More studies are needed to ascertain optimal dosing levels and courses of treatment of ketamine for SUDs. This research is needed to confirm evidence from the OUD and CUD studies suggesting that doses greater than those use in traditional antidepressant ranges, i.e., enough to create psychedelic effects [34,60,61], may offer better efficacy and, from OUD and AUD studies, that repeated sessions are superior to a single administration [40,44]. Most studies reviewed here have examined the effects of ketamine combined with psychotherapies of some kind but some studies from the AUD, CUD, and tobacco use disorder literature hint this may not be necessary [36,37,39,42,62]. Notably, of the RDBCTs, 4/7 did incorporate some form of psychotherapy albeit differently for each trial while 3/7 included no form of psychotherapy and all found preliminary results indicating efficacy. The current evidence leaves the burning unresolved question of whether psychotherapy is essential for ketamine’s treatment of SUDs. At this time, it is unclear what form of psychotherapy is necessary to achieve the largest effect size or to what extent the form or timing of psychotherapy in relation to ketamine administration is significant.

Another unanswered question pertains to dosing format. IV/IM ketamine administration is ubiquitous in the research reviewed here, but this format may not ultimately be ideal. Intranasal esketamine has emerging evidence for efficacy in treating SUDs (see Supplement File S1) and is the only formulation currently supported by FDA approval and a regulatory-safety monitoring system for psychiatric indications [58,64,65]. Notably, while the literature on esketamine for the treatment of SUDs is more limited than that of ketamine, it does have a multicenter, randomized, placebo-controlled trial for the treatment of tobacco use disorder [66] and a double-blind, placebo-controlled pilot study for the treatment of AUD [67], both with positive results. Given that ketamine itself is a recreational drug that can produce dependence and dangerous psychiatric consequences when misused [68], intranasal esketamine may ultimately be better suited for the purpose of treating SUDs [69,70,71,72]. Today’s reality is that ketamine is already being used off-label for the treatment of a variety of psychiatric disorders, including SUDs, in unregulated clinics or at home without oversight. This has raised safety concerns about the development of ketamine use disorder and diversion within these settings [73]. On the other hand, due to the risk evaluation and mitigation strategy program for esketamine, the risk of diversion is relatively low and there has been continuous data collection enabling practitioners to understand the safety profile and long-term outcomes of repeated esketamine administration. Therefore, studies are greatly needed to compare IV/IM ketamine to intranasal esketamine for this purpose.

An additional limitation is the unknown effects of other psychotropic medications participants may have been taking at the time of the study. Multiple trials examined above reference participants with comorbid mental illness but fail to specify if they were taking any other psychotropics during the trial. Besides pharmacokinetic considerations, one is left to wonder if these patient populations responded because of an amplification of ketamine’s mystical experience, synergistic effects on BDNF elevation, enhancement of neuroplasticity, adequate management of psychiatric comorbidities, or by mechanisms yet to be uncovered.

As with other psychedelic drugs being studied for psychiatric indications, a significant challenge in research on ketamine treatment for SUDs is in utilizing appropriate active control medications that can address putative amplification of the placebo effect produced just by experiencing the drug’s acute psychedelic effects. Addressing this conundrum seems particularly important given that available evidence suggests that its efficacy for SUDs might be greater when dosing produces mystical-range psychedelic effects [34,46,60,61]. Both the AUD and CUD research, thus, far has attempted to address this issue with the use of benzodiazepines as a control agent [32,36,37,38], but it is far from certain if this class of drugs is adequate as control for ketamine.

None of the studies reviewed here have identified any major long-term adverse psychiatric effects or the development of SUDs as a result of ketamine delivery within the research context. Although this safety and efficacy data on ketamine for SUDs looks promising, replication of existing data and expansion of rigorous research designs across larger sets of treatment conditions and patients are needed before it can be recommended and accepted for use in routine care of SUDs. However, SUDs remain a devastating and highly lethal diseases of high prevalence in the U.S. and around the world, and this disease burden requires a response and urgency that is proportional to this crisis. Ketamine administered in clinical settings under the supervision of well-trained psychiatric physicians may represent at promising new treatment avenue. Emerging evidence suggests that ketamine can treat a variety of SUDs [28,36,37], with efficacy that lasts longer than its acute intoxicating effects [36,37,39,42,62], while producing minimal to no adverse long-term effects [10,32,33,34,36,37,38,40,41,43,44]. The accumulating data should motivate further expansion of rigorous studies on the applications of ketamine and other drugs with activity on glutamate neurotransmission for SUDs.

## 3. Conclusions

In summary, ketamine has preliminary evidence suggesting its efficacy for SUDs while treating psychiatric comorbidities [35,45] and providing beneficial mystical experiences [10,34,40,43,46,60,61] that may reduce cravings [28,34,36,38,40,45,53], increase abstinence rates [10,32,33,37,42,43,44], and prolong periods of abstinence [10,32,33,34,38,40,43,44]. Growing evidence suggests the drug may have efficacies that span addictions involving different substances, with some data hinting at better efficacy with psychedelic-range dosing [34] and repeated sessions [40,44]. The available research is, however, significantly limited by heterogeneous protocols, a lack of consensus on the best active comparator controls, and many unanswered questions about the importance of timing and form of concurrent psychotherapies for ketamine’s efficacy. There is also very little known about the optimization of drug delivery methods (e.g., as IV or esketamine intranasal preparations), professional supervision, therapeutic environments, and monitoring that must be implemented to ensure safety and protect against misuse in persons with addictions. Given the significant disease burden of SUDs and their massive societal impacts, there is an urgent need for more research to explore the therapeutic potential of ketamine and other drugs active in the brain’s glutamate neurotransmission systems. At present, the available evidence on ketamine is promising enough to warrant much more investigation in appropriately monitored and professionally equipped clinical research settings. However, this literature is still too under-developed to support ketamine’s (or esketamine’s) application specifically for SUDs in routine clinical care, and certainly for its delivery outside of professionally supervised psychiatric treatment settings.

## Figures and Tables

**Table 1:** Characteristics of RDBCTs included.

Author, Year	Substance	Study Design	Participants	Intervention	Psychotherapy	Outcomes	Conclusion
Dakwar et al., 2020 [32]	Alcohol	RDBCT	40 adults with alcohol dependence	KET (0.71 mg/kg), MDZ (0.025 mg/kg)	MET	Drinking days during 21 days post-infusion	A single KET infusion alongside MET decreased alcohol consumption
Grabski et al., 2022 [33]	Alcohol	RDB Phase 2 CT	96 adults with moderate to severe AUD	KET (0.8 mg/kg) + MBRP, KET + AE, PBO + MBRP, PBO + AE	MBRP or AE	Self-reported percentage days abstinent at 6 months post-first-infusion, Confirmed relapse at 6 months post-first-infusion	Three KET infusions and MBRP led to greater percentage of days abstinent than PBO and AE
Krupitsky et al., 2002 [34]	Opioid	RDBCT	70 inpatient adults with heroin addiction	Psychedelic dosing KET (2.0 mg/kg), Non-psychedelic dosing KET (0.2 mg/kg)	KPT	Abstinence over 24 months post-infusion, Cravings over 24 months post-infusion	Psychedelic dosing of KET may be superior to non-psychedelic dosing in improving abstinence and decreasing cravings 24 months post-infusion
Mansoori et al., 2025 [35]	Opioid	RDBCT	64 inpatient adults with MDD and OUD	KET (0.5 mg/kg), BUP (16 mg)	None	Anxiety over 1 week post-intervention, Craving over 1 week post-intervention	KET was as effective as BUP in reducing anxiety and opioid cravings
Dakwar et al., 2014 [36]	Cocaine	CRDBT	8 non-treatment-seeking adults with cocaine dependence	Three infusions in random order: KET (0.41 mg/kg), KET (0.71 mg/kg), LZP (2 mg)	None	Motivation to quit cocaine 24 h post-infusion, Cue-induced craving 24 h post-infusion	KET reduced cue-induced craving and increased motivation to quit with higher efficacy at higher dose
Dakwar et al., 2016 [37]	Cocaine	CRDBT	20 non-treatment-seeking adults with cocaine dependence	KET (0.71 mg/kg), MDZ (0.025 mg/kg)	None	Cocaine self-administration	KET led to a reduction in cocaine self-administration
Dakwar et al., 2019 [38]	Cocaine	RDBCT	55 adults with cocaine dependence	KET (0.5 mg/kg), MDZ (0.025 mg/kg)	MBRP	2 weeks of end-of-study abstinence	A single KET infusion alongside MBRP increased abstinence, decreased craving, and prolonged time to relapse

Abbreviations: KET: Ketamine, PBO: Placebo, RDBCT: Randomized, Double-Blind, Controlled Trial, MDZ: Midazolam, MET: Motivational Enhancement Therapy, RDB: Randomized, Double-Blind, CT: Clinical Trial, AUD: Alcohol Use Disorder, MBRP: Mindfulness-Based Relapse Prevention, AE: Alcohol Education, KPT: Ketamine Psychotherapy, MDD: Major Depressive Disorder, OUD: Opioid Use Disorder, BUP: Buprenorphine, CRDBT: Crossover, Randomized, Double-Blind Trial, LZP: Lorazepam.

**Table 2:** Characteristics of other key included studies.

Author, Year	Substance	Study Design	Participants	Intervention	Psychotherapy	Outcomes	Conclusion
Das et al., 2019 [28]	Alcohol	SBRCT	90 non-treatment-seeking adults with hazardous drinking	RET + KET (serum 350 ng/mL for 30 min), no RET + KET, RET + PBO	None	Reactivity to alcohol and alcohol cues, Changes in alcohol consumption	KET may disrupt the reconsolidation of maladaptive reward memories and thus decrease alcohol consumption
Terasaki et al., 2022 [39]	Alcohol	3-Arm, Open-Label, RCT	44 hospitalized adults with severe AUD	KET (0.5 mg/kg), IM NTX, LA	None	All-cause, 30-day hospital readmission rate	A single KET infusion or IM NTX show promise in reducing the readmission rate
Krupitsky et al., 2007 [40]	Opioid	RCT	59 inpatient adults with heroin dependence	1 KET (2.0 mg/kg) infusion, 3 KET infusions	KPT	Abstinence over 12 months post-infusion(s)	Repeated KET infusions may be superior to a single KET infusion in improving abstinence 12 months post-last-infusion
Azhari et al., 2021 [41]	Cannabis	Single-Blind, Proof Of Concept Trial	8 adults with cannabis dependence	KET (0.71 mg/kg), non-responders also received KET (1.41 mg/kg)	MET and MBRP	Number of cannabis-using days per week	KET infusion(s) resulted in less cannabis-using days per week
Chuang et al., 2025 [42]	Tobacco	SBRCT	10 non-treatment-seeking adults with tobacco use disorder	KET (0.5 mg/kg)	None	Tobacco use, Tobacco craving, Tobacco withdrawal	A single KET infusion shows promise in reducing tobacco craving and withdrawal

Abbreviations: SBRCT: Single-Blind Randomized Controlled Trial, KET: Ketamine, RET: Retrieval of Alcohol-Maladaptive Reward Memories, PBO: Placebo, MET: Motivational Enhancement Therapy, AUD: Alcohol Use Disorder, MBRP: Mindfulness-Based Relapse Prevention, RCT: Randomized Controlled Trial, NTX: Naltrexone, LA: Linkage Alone, KPT: Ketamine Psychotherapy.

## Data Availability

No new data was created or analyzed in this study. Data sharing is not applicable to this article.

## Supplementary Materials

The following supporting information can be downloaded at: https://www.mdpi.com/article/10.3390/neurosci6030083/s1, File S1: Esketamine’s Therapeutic Role in Substance Use Disorders.
