# Supplementary material for: Ketamine’s Therapeutic Role in Substance Use Disorders: A Narrative Review
Source: NeuroSci. 2025 Aug 27;6(3):83. doi: 10.3390/neurosci6030083 (PMC12452417; doi:10.3390/neurosci6030083)
Supplement: Supplementary file 1 [file neurosci-06-00083-s001.zip › neurosci-3790759-supplementary.pdf]

NeuroSci

MDPI

Supplementary Materials:

Supplement S1.

Esketamine currently has one double-blind pilot study in support of treating alcohol use disorder (AUD) [67], preclinical evidence for the treatment of opioid use disorder (OUD) and cocaine use disorder [69,70], and a multicenter, randomized, placebo-controlled study in support of treating tobacco use disorder [66]. Additionally, two studies examined the reporting odds ratios for esktamine from the Food and Drug Administration adverse event reporting system (FAERS) and the World Health Organization pharmacovigilance database which showed some associations between esketamine and lower reporting odds ratios of substance use disorders [71,72].

The double-blind, placebo-controlled, pilot study by Gent et al. where 28 adults with either hazardous drinking (15) or moderate to severe alcohol use disorder (13) where randomized in a 1:1 ratio to either receive sublingual esketamine (115.1 mg) or sublingual vitamin C once on day 8. For 7 days before and after drug administration, they were given daily mindfulness practice with the final follow up occurring 14 days after the initial visit. The esketamine group showed more engagement with the mindfulness-based intervention than placebo and significantly less post-administration craving. Both groups significantly decreased alcohol consumption and increases in mindfulness [67].

We know of no human studies examining esketamine for the treatment of OUD or cocaine use disorder in humans. Thus, two relevant preclinical studies are presented. With regards to OUD, Fontoura et al. conducted a study on 32 male Wistar rats to see if esketamine administered daily for 10 days or twice every 5 days altered condition place preference for morphine. The morphine conditioned place preference was extinguished in both groups after a period of abstinence suggesting that esketamine administration could be beneficial in reducing the rates of relapse within the context of OUD [69]. With regards to cocaine use disorder, Wydra et al. conducted a study on 39 male Wistar rats administered esketamine once to examine if cocaine-seeking behavior would decrease. The rats significantly decreased their cocaine-seeking behavior as shown by a decrease in the number of active lever (cocaine administration) presses after esketamine administration [70].

Hong et al. conducted a multicenter, randomized, placebo-controlled clinical trial for patients with major depressive disorder (MDD), lung cancer, and tobacco use disorder to investigate if 8 sessions of esketamine improved the rates of both self-reported and biologically verified rates of smoking abstinence. Two hundred thirty six current daily cigarette smoking adults with a recent diagnosis of lung cancer and MDD with two failed treatment trials underwent weekly intranasal esketamine administration with monthly follow-ups for 6 months to determine smoking abstinence status. There was a significantly higher abstinence rate in the esketamine group as compared to placebo alongside significantly more improvement in depression and anxiety scales [66].

Kwan et al. first examined post-marketing adverse events reported to the FDA in their database FAERS. Using acetaminophen as a control, they calculated the reporting odds ratio (ROR) representing the odds of the occurrence of an adverse event, in this case alcohol or substance use disorders, associated with exposure to ketamine and esketamine. For esketamine, the RORs were significantly reduced for substance abuse, drug dependence, and drug abuse suggesting an association with lower rates of these disorders while acknowledging no causality can be implied by such study [71]. The same group performed a replication study using the World Health Organization pharmacovigilance database. They again found that esketamine had significantly lower RORs for substance abuse, drug dependence, and drug abuse reinforcing their initial conclusion [72].
